# Dysregulated transient receptor potential channel 1 expression and its correlation with clinical features and survival profile in surgical non‐small‐cell lung cancer patients

**DOI:** 10.1002/jcla.24229

**Published:** 2022-02-02

**Authors:** Changjiang Ke, Shenghua Long

**Affiliations:** ^1^ Department of Respiratory and Critical Care Medicine (Respiratory Medicine) Huangshi Central Hospital Affiliated Hospital of Hubei Polytechnic University Edong Healthcare Group Hubei China; ^2^ Hubei Key Laboratory of Kidney Disease Pathogenesis and Intervention Hubei China

**Keywords:** disease‐free survival, lymph node metastasis, non‐small‐cell lung cancer, overall survival, transient receptor potential channel 1

## Abstract

**Background:**

Transient receptor potential channel 1 (TRPC1) facilitates the tumor growth, metastasis, and chemoresistance in a series of neoplasms, while its correlation with clinical features and survival profile in NSCLC patients remains elusive. Hence, this study aimed to explore this topic.

**Methods:**

Totally, 192 NSCLC patients were enrolled. Protein and mRNA expression of TRPC1 in carcinoma tissue and para‐carcinoma tissue were evaluated by immunohistochemistry (IHC) assay and reverse transcription quantitative polymerase chain reaction (RT‐qPCR) assay, respectively.

**Results:**

Immunohistochemistry score and mRNA expression of TRPC1 were higher in carcinoma tissue compared with para‐carcinoma tissue (both *p *< 0.001). Besides, increased TRPC1 IHC score (*p* = 0.004) and elevated TRPC1 mRNA overexpression (*p* = 0.016) were linked with occurrence of LYN metastasis; meanwhile, increased TRPC1 IHC score (*p* = 0.015) and raised TRPC1 mRNA expression (*p* = 0.009) were also linked with advanced TNM stage, whereas TRPC1 IHC score and TRPC1 mRNA expression were not correlated with other clinical features (all *p >* 0.05). Additionally, TRPC1 protein high (*p* = 0.007) and TRPC1 mRNA high (*p* = 0.015) were correlated with poor disease‐free survival (DFS) but not correlated with overall survival (OS). Moreover, multivariate Cox's proportional hazards regression analysis showed that high TRPC1 protein expression (*p* = 0.046) and advanced TNM stage (*p *< 0.001) were independently correlated with poor DFS. However, TRPC1 protein and mRNA expression were not linked with OS (both *p* > 0.05), while poor differentiation (*p* = 0.003) and advanced TNM stage (*p *< 0.001) were independently associated with worse OS.

**Conclusions:**

TRPC1 is unregulated in NSCLC tissue with its overexpression relating to the occurrence of LYN metastasis and worse DFS in NSCLC patients.

## Graphical abstract

Totally, 192 non‐small‐cell lung cancer (NSCLC) patients were enrolled. TRPC1 protein expression in 192 pairs of carcinoma tissue and para‐carcinoma tissue was evaluated by IHC assay; meanwhile, TRPC1 mRNA expression in 110 pairs of carcinoma tissue and para‐carcinoma tissue was evaluated by RT‐qPCR assay. IHC score and mRNA expression of TRPC1 were higher in carcinoma tissue compared with para‐carcinoma tissue (both *p* < 0.001). Besides, increased TRPC1 IHC score (*p* = 0.004) and elevated TRPC1 mRNA overexpression (*p* = 0.016) were linked with the occurrence of LYN metastasis. Additionally, TRPC1 protein high (*p* = 0.007) and TRPC1 mRNA high (*p* = 0.015) were correlated with poor DFS of NSCLC patients.

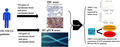

## 1 INTRODUCTION

Lung cancer is one of the main causes of cancer‐related death worldwide with an estimated 2.2 million new cancer cases and 1.8 million deaths in 2020, among which 85% cases are diagnosed as non‐small‐cell lung cancer (NSCLC).^1^, ^2^ With the development of novel drug and the realization of precise medicine, the outcome of NSCLC is improved to some extent.^3^, ^4^ Unfortunately, a nonnegligible proportion of NSCLC patients still cannot respond to the current therapy with an unfavorable five‐year survival, which ranges from 4% to 17%.^5^, ^6^ Hopefully, accumulative studies have demonstrated the prognostic potential of new biomarkers for NSCLC. To be specific, accumulating researches disclose that a series of hallmarks such as miR‐155, CDK4, and MMP‐14 are selected for monitoring disease progression and prognostication,^7^, ^8^, ^9^ whereas this issue still needs further investigation; thus, the significance of detecting novel hallmarks for NSCLC should be emphasized.

Transient receptor potential channel 1 (TRPC1), a family member of non‐selective cation channel proteins, mediates store‐operated calcium entry.^10^, ^11^ Besides, accumulating evidences illustrate that TRPC1 may have an oncogenic potential, which facilitates the proliferation, invasion, and dedifferentiation in numerous carcinomas.^10^, ^11^ More importantly, several studies illuminate that TRPC1 is highly expressed and shows prognostic value in some carcinomas.^12^, ^13^ Detailly, TRPC1 is upregulated in breast cancer tissue and is correlated with advanced clinicopathological features as well as poor prognosis in breast cancer patients.^14^, ^15^ Likewise, TRPC1 is overexpressed and links with unsatisfying clinicopathological characteristics or poor prognosis in patients with colorectal cancer and gastric cancer.^16^, ^17^, ^18^ However, its clinical involvement in NSCLC remains elusive. Hence, the current study aimed to investigate the aberrant expression of TRPC1 and its relation to clinical features and survival profile in NSCLC patients.

## 2 MATERIALS AND METHODS

### 2.1 Participants

With the approval of the Institutional Review Board of Huangshi Central Hospital, Affiliated Hospital of Hubei Polytechnic University, Edong Healthcare Group, this retrospective study reviewed 192 NSCLC patients who received surgical resection in our hospital from January 2016 to December 2019. The screening criteria for eligible patients were: (a) diagnosis of NSCLC by histopathological examinations; (b) TNM stage I‐III; (c) treated by radical operation; (d) with available formalin‐fixed paraffin‐embedded (FFPE) samples to conduct immunohistochemistry (IHC) assay; (e) with available clinical data and survival data. The patients were ineligible for the study if they had the conditions as follows: (a) history of other cancers or malignancies at diagnosis; (b) history of systemic therapy prior to radical operation; and (c) no any follow‐up records.

### 2.2 Data collection

Clinical data were abstracted from the medical records, which included demographics, comorbidities, tumor markers, surgical information, and adjuvant chemotherapy regimens. Surgical resection was operated for the corresponding patients according to the disease status: for TNM stage I‐II patients, pulmonary lobectomy plus lymph node dissection or pneumonectomy plus lymph node dissection was performed; for TNM stage III patients, pulmonary lobectomy, pneumonectomy, or lymph node dissection were performed. A total of 135 patients received adjuvant chemotherapy as disease requirement, and adjuvant chemotherapy regimens comprised of navelbine+cisplatin (NP), docetaxel+cisplatin or carboplatin (DP), taxol+cisplatin or carboplatin (TP), and gemcitabine+cisplatin or carboplatin (GP). In addition, the follow‐up data were collected to calculate the disease‐free survival (DFS) and overall survival (OS), with a deadline of June 30, 2021.

### 2.3 Sample collection

All 192 patients' FFPE samples of carcinoma tissue and para‐carcinoma tissue were collected to evaluate the expression of TRPC1 protein by IHC assay. Meanwhile, 110 of 192 patients also had fresh‐frozen carcinoma tissue and para‐carcinoma tissue samples, which were obtained to determine the expression of TRPC1 mRNA by reverse transcription quantitative polymerase chain reaction (RT‐qPCR) assay.

### 2.4 IHC assay

The procedure of IHC assay was in accord with a previous study.^19^ In brief, the goat polyclonal anti‐TRPC1 antibody (1:150; Abcam) was applied as primary antibody, and the rabbit anti‐goat IgG (H&L) (1:2000; Abcam) was applied as secondary antibody. The experiment was carried out in strict accordance with the instructions. After IHC assay, the expression of TRPC1 protein was assessed by semi‐quantitatively method based on the intensity and density of positive carcinoma cells. IHC staining intensity was classified as four grades: 0 (negative), 1 (weak), 2 (moderate), and 3 (strong). IHC staining density was classified as five grades: 0 (0%), 1 (1%‐25%), 2 (26%‐50%), 3 (51%‐75%), and 4 (76%‐100%). The max IHC score was 12 which was a product of two above staining scores.

### 2.5 RT‐qPCR assay

RT‐qPCR was performed to evaluate the TRPC1 mRNA expression. The total RNA was extracted by TRIzol™ Reagent (Thermo Fisher Scientific). RNA was reversely transcribed to cDNA using iScript™ cDNA Synthesis Kit (with random primer; Bio‐Rad). Moreover, qPCR was performed using QuantiNova SYBR Green PCR Kit (Qiagen). Primers were designed in accordance with a previous study.^20^ Glyceraldehyde‐3‐phosphate dehydrogenase (GAPDH) was used as internal references; meanwhile, TRPC1 expression was calculated using the 2^–ΔΔCt^ method.

### 2.6 Definition

According to the total IHC score, the expression of TRPC1 protein was categorized as high expression (IHC score >3) and low expression (IHC score ≤3). According to the result of RT‐qPCR assay, the expression of TRPC1 mRNA was classified as high expression (>2.268) and low expression (≤2.268) by its median value (2.268) in carcinoma tissue.

### 2.7 Statistical analysis

SPSS 26.0 (IBM Corp.) was used for data analyses, and GraphPad Prism 7.02 (GraphPad Software Inc.) was applied for graph constructions. Paired‐samples t test and Wilcoxon signed‐rank test were used to compare the TRPC1 expression between carcinoma tissue and para‐carcinoma tissue, and receiver‐operating characteristic (ROC) curve analysis was performed to estimate the performance of TRPC1 expression in distinguishing different tissue. Correlation between TRPC1 expression and clinical characteristics was determined by Student's *t* test, Mann–Whitney *U* test, one‐way analysis of variance (ANOVA) test, Kruskal–Wallis *H* rank sum test, and Spearman's rank correlation test as appropriate. DFS and OS were elucidated using Kaplan–Meier curves and compared by log‐rank test. Cox's proportional hazard regression was carried out for prognostic factor analysis, and hazard ratio with 95% confidence intervals (CI) was displayed using forest plot. All factors (including TRPC1 protein (high vs. low), TRPC1 mRNA (high vs. low), age, gender (male vs. female), history of smoking (yes vs. no), history of drinking (yes vs. no), history of hypertension (yes vs. no), history of hyperlipidemia (yes vs. no), history of diabetes (yes vs. no), histological subtypes (including ASC, ADC, and SCC), poor differentiation, tumor size (>5 cm vs. ≤5 cm), LYN metastasis (yes vs. no), high TNM stage, ECOG PS score (1 vs. 0), CEA (>5 ng/mL vs. ≤5 ng/mL), CA125 (>35 U/mL vs. ≤35 U/mL), and adjuvant chemotherapy (yes vs. no)) were incorporated in multivariable analysis using forward stepwise (conditional LR) method to screen out independent prognostic factors. All tests were two‐sided, and *p* < 0.05 indicated a statistical significance.

## 3 RESULTS

### 3.1 Clinical characteristics

Totally, 192 NSCLC patients were analyzed with a mean age of 61.9 ± 11.6 years (Table 1). In terms of gender, there were 33 (17.2%) females and 159 (82.8%) males. Moreover, 110 (57.3%) and 68 (35.4%) patients had a history of smoking and drinking, separately. Additionally, 28 (14.6%), 106 (55.2%) and 58 (30.2%) patients had well, moderate, and poor differentiation tumor. Besides, the median tumor size was 5.5 (interquartile range (IQR) 4.0–8.0) cm. In regard to TNM stage, there were 43 (22.4%), 83 (43.2%), and 66 (34.4%) patients diagnosed as stage I, stage II, and stage III. What's more, there were 149 (77.6%) and 43 (22.4%) patients with ECOG PS score 0 and ECOG PS score 1, separately. Beyond that, 80 (41.7%), 23 (12.0%), 16 (8.3%), and 16 (8.3%) patients received NP, DP, TP, and GP adjuvant regimen, respectively, while 57 (29.7%) patients did not receive adjuvant chemotherapy. More detailed information is listed in Table 1.

**TABLE 1:** Clinical characteristics

Items	NSCLC patients (*N* = 192)
**Demographics**	
Age (years), mean ± SD	61.9±11.6
Gender, no. (%)	
Female	33 (17.2)
Male	159 (82.8)
History of smoking, no. (%)	110 (57.3)
History of drinking, no. (%)	68 (35.4)
**Comorbidities**	
History of hypertension, no. (%)	59 (30.7)
History of hyperlipidemia, no. (%)	64 (33.3)
History of diabetes, no. (%)	32 (16.7)
**Disease characteristics**	
Histological subtype, no. (%)	
ADC	98 (51.0)
SCC	78 (40.6)
ASC	16 (8.3)
Differentiation, no. (%)	
Well	28 (14.6)
Moderate	106 (55.2)
Poor	58 (30.2)
Tumor size (cm), median (IQR)	5.5 (4.0–8.0)
LYN metastasis, no. (%)	71 (37.0)
TNM stage, no. (%)	
I	43 (22.4)
II	83 (43.2)
III	66 (34.4)
ECOG PS score, no. (%)	
0	149 (77.6)
1	43 (22.4)
**Tumor markers**	
CEA (ng/mL), median (IQR)	5.8 (2.5–33.3)
CA125 (U/mL), median (IQR)	34.4 (13.4–77.8)
**Adjuvant chemotherapy**	
No, no. (%)	57 (29.7)
Yes, no. (%)	135 (70.3)
NP regimen	80 (41.7)
DP regimen	23 (12.0)
TP regimen	16 (8.3)
GP regimen	16 (8.3)

Abbreviations: ADC, adenocarcinoma; ASC, adenosquamous carcinoma; CA125, cancer antigen 125; CEA, carcinoembryonic antigen; DP, docetaxel+cisplatin or carboplatin; ECOG PS, Eastern Cooperative Oncology Group performance status; GP, gemcitabine+cisplatin or carboplatin; IQR, interquartile range; LYN, lymph node; NP, navelbine+cisplatin; NSCLC, non‐small‐cell lung cancer; SCC, squamous cell carcinoma; SD, standard deviation; TNM, tumor‐node‐metastasis; TP, taxol+cisplatin or carboplatin.

### 3.2 Protein and mRNA expression of TRPC1

**FIGURE 1:**
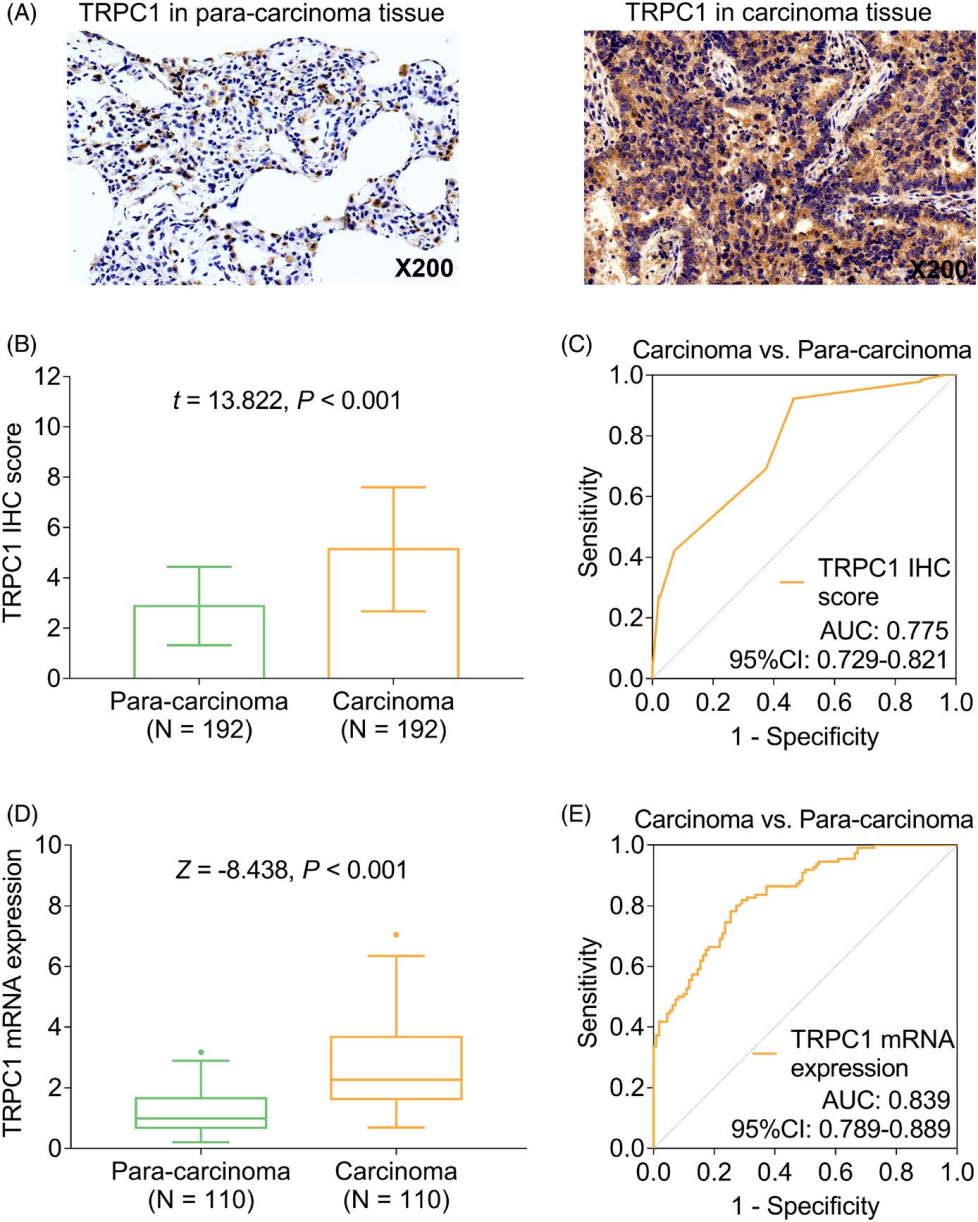
Expression of TRPC1 in carcinoma tissue and para‐carcinoma tissue. Detecting TRPC1 protein expression by IHC assay (A); comparison of TRPC1 IHC score between carcinoma tissue and para‐carcinoma tissue (B); ROC curve of TRPC1 IHC score in distinguishing carcinoma from para‐carcinoma (C); comparison of TRPC1 mRNA expression between carcinoma tissue and para‐carcinoma tissue (D); ROC curve of TRPC1 mRNA expression in distinguishing carcinoma from para‐carcinoma (E). TRPC1: transient receptor potential channel 1; NSCLC: non‐small‐cell lung cancer; IHC: immunohistochemistry; AUC, area under curve

TRPC1 protein expression in carcinoma tissue as well as para‐carcinoma tissue was detected by IHC assay (Figure 1A). To be specific, TRPC1 IHC score was higher in carcinoma tissue compared with para‐carcinoma tissue (*t* = 13.822, *p* < 0.001, Figure 1B); meanwhile, ROC analysis disclosed that TRPC1 IHC score exhibits a good capability in distinguishing carcinoma tissue from para‐carcinoma tissue (AUC: 0.775, (95% confidence interval (CI): 0.729–0.821), Figure 1C). Besides, TRPC1 mRNA expression was higher in carcinoma tissue compared with para‐carcinoma tissue (*Z* = −8.438, *p* < 0.001, Figure 1D) as well; meanwhile, TRPC1 mRNA expression exhibited a good capability in distinguishing carcinoma tissue from para‐carcinoma tissue (AUC: 0.839; 95% CI: 0.789–0.889; Figure 1E).

### 3.3 Correlation of TRPC1 expression with clinical features

**FIGURE 2:**
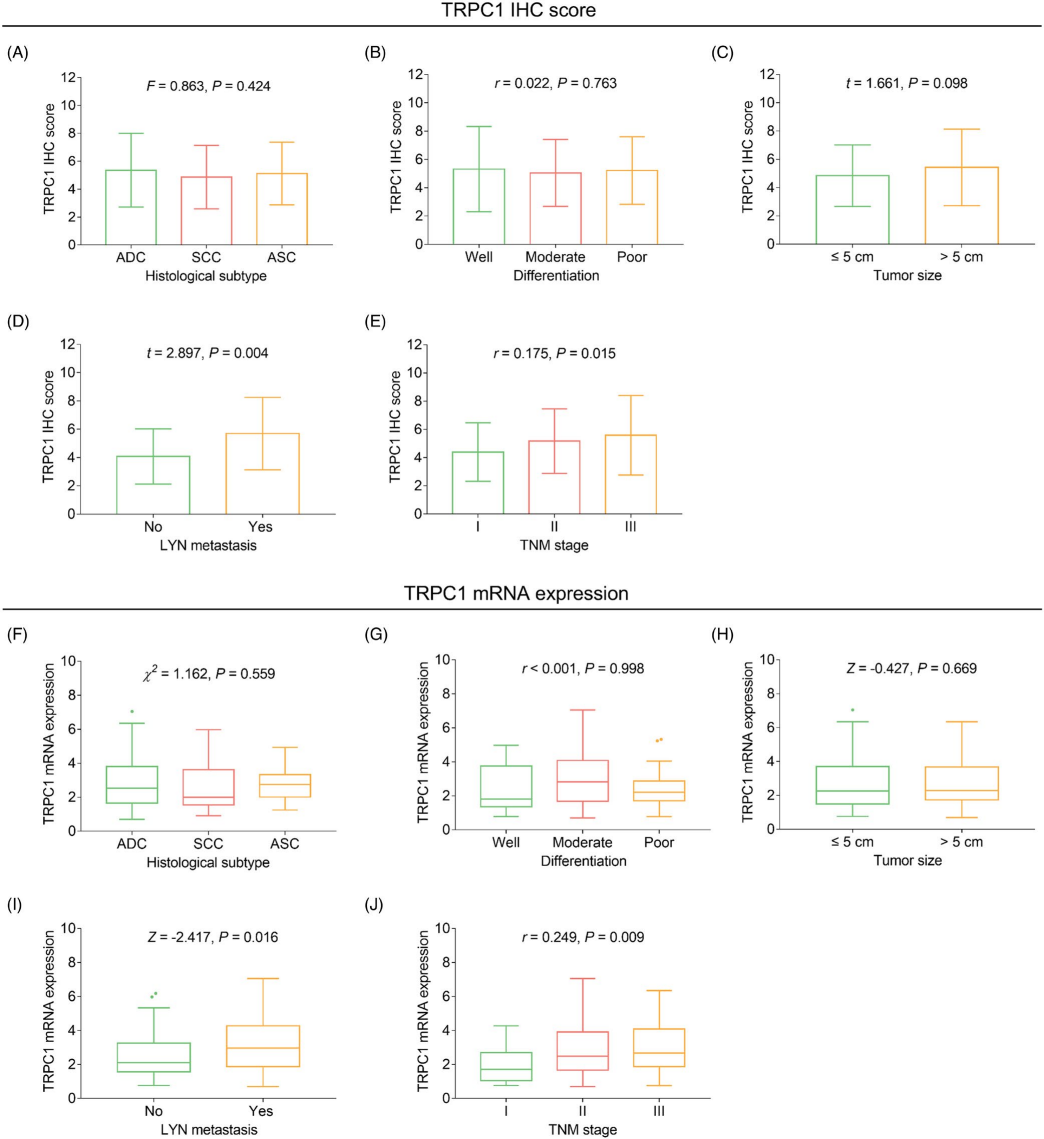
Association between TRPC1 expression and clinicopathological characteristics. Association of TRPC1 IHC score with histological subtype (A), differentiation (B), tumor size (C), LYN metastasis (D), and TNM stage (E); association of TRPC1 mRNA expression with histological subtype (F), differentiation (G), tumor size (H), LYN metastasis (I), and TNM stage (J). TRPC1: transient receptor potential channel 1; IHC: immunohistochemistry; TNM: tumor‐node‐metastasis; LYN, lymph node metastasis

Elevated TRPC1 IHC score was correlated with the occurrence of LYN metastasis (*t* = 2.897, *p* = 0.004); meanwhile, increased TRPC1 IHC score was also correlated with advanced TNM stage (*r* = 0.175, *p* = 0.015). Likewise, elevated TRPC1 mRNA expression was correlated with the occurrence of LYN metastasis (*Z* = −2.417, *p* = 0.016) and advanced TNM stage (*r* = 0.249, *p* = 0.009), whereas TPRC expression was not correlated with other clinical features (all *p* > 0.05, Figure 2A–H). Additionally, TRPC1 mRNA and protein expressions were not linked with tumor markers (all *p* > 0.05, Table S1).

### 3.4 Correlation of TRPC1 expression with survival

**FIGURE 3:**
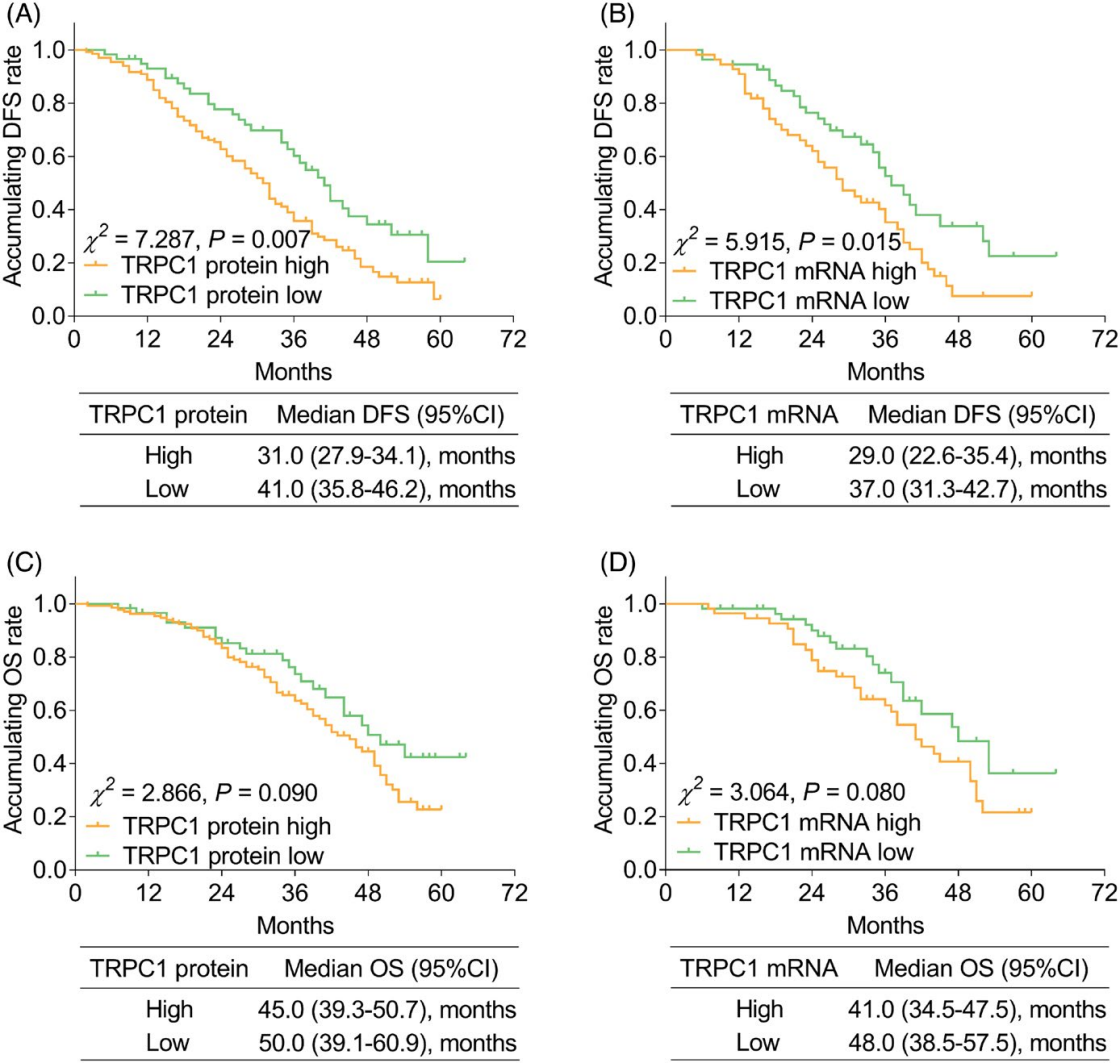
Association betweenTRPC1 expression and accumulating EFS and OS. Correlation of TRPC1 protein level (A) and TRPC1 mRNA expression (B) with accumulating DFS; correlation of TRPC1 protein level (C); and TRPC1 mRNA expression (D) with accumulating OS. DFS: disease‐free survival; OS: overall survival; TRPC1, transient receptor potential channel 1

TRPC1 protein high (*χ* ^2^ = 7.287, *p* = 0.007, Figure 3A) and TRPC1 mRNA high (*χ* ^2^ = 5.915, *p* = 0.015, Figure 3B) were both correlated with unfavorable accumulating DFS. However, TRPC1 protein (*χ* ^2^ = 2.866, *p* = 0.090, Figure 3C) and TRPC1 mRNA (*χ* ^2^ = 3.064, *p* = 0.080, Figure 3D) were not correlated with accumulating OS.

Additionally, subgroup analysis was performed on account of whether patients underwent adjuvant therapy, which illuminated that TRPC1 protein high (*χ* ^2^ = 3.950, *p* = 0.047, Figure S1A) and TRPC1 mRNA high (*χ* ^2^ = 4.674, *p* = 0.031, Figure S1b) were linked with poor accumulating DFS in patients undergoing adjuvant chemotherapy, whereas other subgroup analysis revealed that there was no more correlation between TRPC1 expression and survival profiles (Figure S1C–H).

### 3.5 Independent factors associated with survival

**FIGURE 4:**
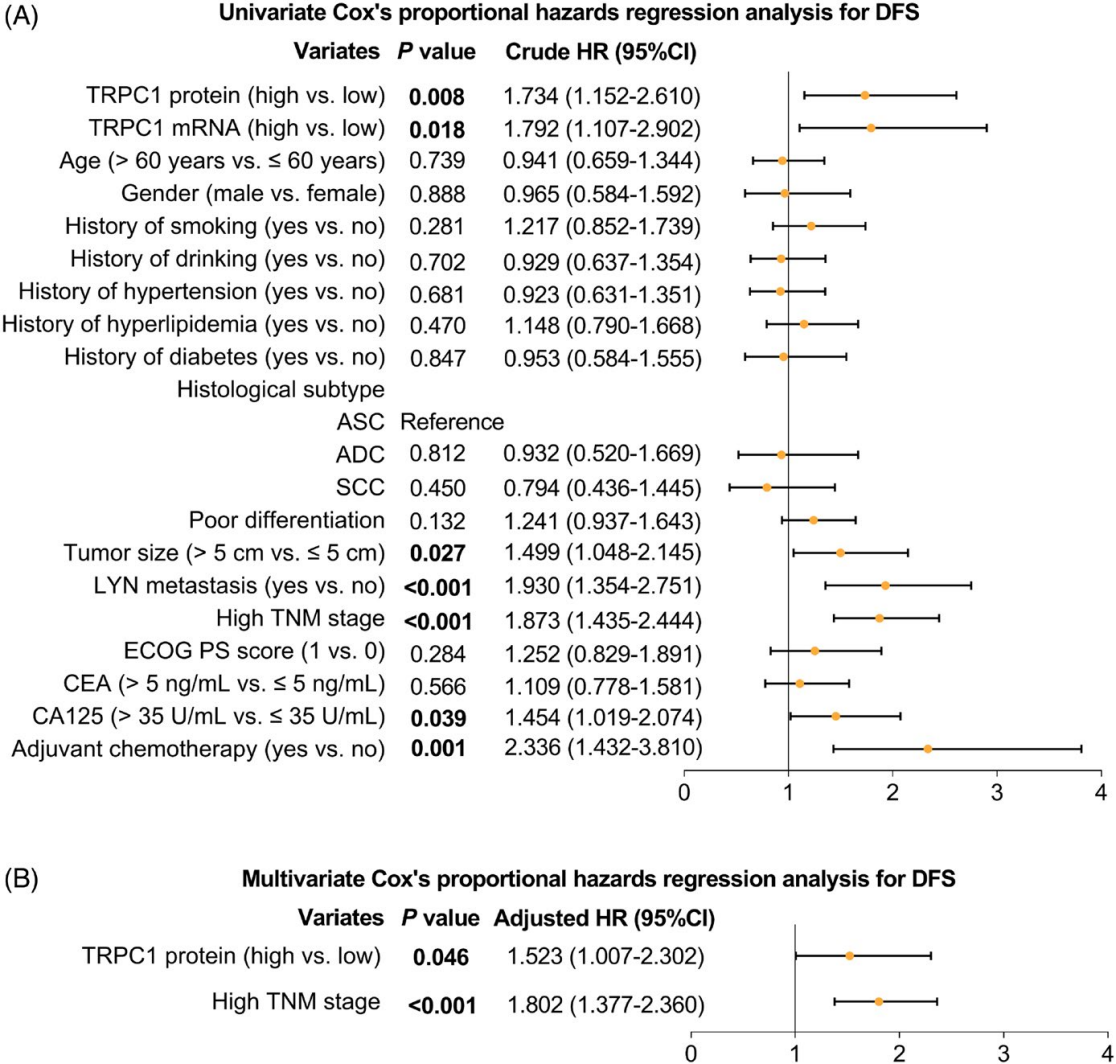
Factors associated with DFS. Univariate (A) and multivariate (B) Cox's proportional hazards regression analysis for DFS (All factors (including TRPC1 protein (high vs. low), TRPC1 mRNA (high vs. low), age, gender (male vs. female), history of smoking (yes vs. no), history of drinking (yes vs. no), history of hypertension (yes vs. no), history of hyperlipidemia (yes vs. no), history of diabetes (yes vs. no), histological subtypes (including ASC, ADC, and SCC), poor differentiation, tumor size (>5 cm vs. ≤5 cm), LYN metastasis (yes vs. no), high TNM stage, ECOG PS score (1 vs. 0), CEA (>5 ng/mL vs. ≤5 ng/mL), CA125 (>35 U/mL vs. ≤35 U/mL), and adjuvant chemotherapy (yes vs. no)) were incorporated in multivariable analysis using forward stepwise (conditional LR) method to screen out independent prognostic factors). DFS: disease‐free survival; TRPC1, transient receptor potential channel 1; TNM, tumor‐node‐metastasis; LYN, lymph node metastasis; CEA, carcinoembryonic antigen; CA125, cancer antigen 125; ECOG PS, Eastern Cooperative Oncology Group performance status; ADC, adenocarcinoma; SCC, squamous cell carcinoma; ASC, adenosquamous carcinoma; HR, hazard ratio; CI, confidence interval

Univariate Cox's proportional hazards regression analysis for DFS revealed that TRPC1 protein high (crude hazard ratio (HR): 1.734, *p* = 0.008), TRPC1 mRNA high (crude HR: 1.792, *p* = 0.018), tumor size >5 cm (crude HR: 1.499, *p* = 0.027), the occurrence of LYN metastasis (crude HR: 1.930, *p* < 0.001), high TNM stage (crude HR: 1.873, *p* < 0.001), CA125 > 35 U/mL (crude HR: 1.454, *p* = 0.039), and adjuvant chemotherapy (crude HR: 2.336, *p* = 0.001) were correlated with worse DFS (Figure 4A). Meanwhile, multivariate Cox's proportional hazards regression analysis for DFS uncovered that TRPC1 protein high (adjusted HR: 1.523, *p* = 0.046) and high TNM stage (adjusted HR: 1.802, *p* < 0.001) were independently correlated with poorer DFS (Figure 4B).

**FIGURE 5:**
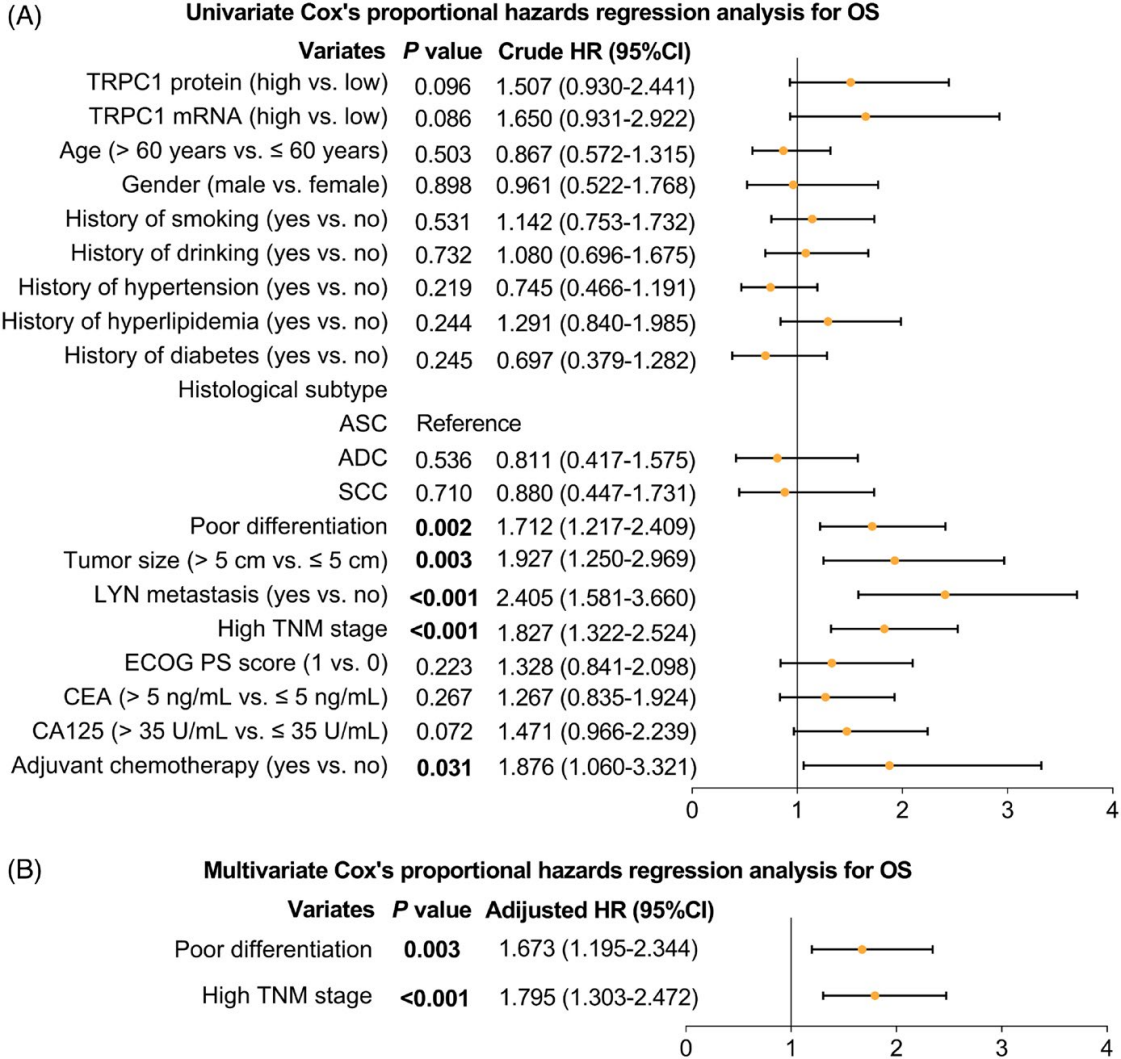
Factors associated with OS. Univariate (A) and multivariate (B) Cox's proportional hazards regression analysis for OS (All factors (including TRPC1 protein (high vs. low), TRPC1 mRNA (high vs. low), age, gender (male vs. female), history of smoking (yes vs. no), history of drinking (yes vs. no), history of hypertension (yes vs. no), history of hyperlipidemia (yes vs. no), history of diabetes (yes vs. no), histological subtypes (including ASC, ADC, and SCC), poor differentiation, tumor size (>5 cm vs. ≤5 cm), LYN metastasis (yes vs. no), high TNM stage, ECOG PS score (1 vs. 0), CEA (>5 ng/mL vs. ≤5 ng/mL), CA125 (>35 U/mL vs. ≤35 U/mL), and adjuvant chemotherapy (yes vs. no)) were incorporated in multivariable analysis using forward stepwise (conditional LR) method to screen out independent prognostic factors). OS, overall survival; TRPC1, transient receptor potential channel 1; TNM, tumor‐node‐metastasis; LYN, lymph node metastasis; CEA, carcinoembryonic antigen; CA125, cancer antigen 125; ECOG PS, Eastern Cooperative Oncology Group performance status; ADC, adenocarcinoma; SCC, squamous cell carcinoma; ASC, adenosquamous carcinoma; HR, hazard ratio; CI, confidence interval

Meanwhile, univariate Cox's proportional hazards regression analysis for OS disclosed that poor differentiation (crude HR: 1.712, *p* = 0.002), tumor size >5 cm (crude HR: 1.927, *p* = 0.003), the occurrence of LYN metastasis (crude HR: 2.405, *p* < 0.001), high TNM stage (crude HR: 1.827, *p* < 0.001), and adjuvant chemotherapy (crude HR: 1.876, *p* = 0.031) were correlated with worse OS (Figure 5A). Moreover, multivariate Cox's proportional hazards regression analysis for OS illuminated that poor differentiation (adjusted HR: 1.673, *p* = 0.003) and high TNM stage (adjusted HR: 1.795, *p* < 0.001) were independently associated with worse OS (Figure 5B).

## 4 DISCUSSION

TRPC1 serves as an iron channel facilitating Ca^2+^ entry in the store‐operated Ca^2+^ entry (SOCE) pathway.^10^, ^11^ More importantly, TRPC1 acts as a tumor promoter by initiating tumor growth and metastasis as well as facilitating tumor chemoresistance.^21^, ^22^, ^23^, ^24^, ^25^, ^26^, ^27^, ^28^, ^29^, ^30^, ^31^ In detail: (1) TRPC1 facilitates tumor cell proliferation by arresting the transition of tumor cell from G1 phase to S phase^23^ (2). TRPC1 facilitates the Ca^2+^ entry in tumor cells; furthermore, Ca^2+^ influx is closely linked with tumor cell migration and metastasis through mediating orchestrating cytoskeletal recombination, focal adhesions, and front–rear end polarity.^24^, ^25^, ^26^, ^27^, ^28^, ^29^, ^30^ Correspondingly, TRPC1 facilitates the tumor cell migration or metastasis. (3) TRPC1 facilitates the autophagy and further promotes the survival, growth, and metastasis of tumor cells by repressing the phosphorylation of the cyclin‐dependent kinase and inducing cyclin E expressions.^21^, ^22^ (4) TRPC1 induces the Ca^2+^ influx and further conduces to the resistance of tumor cells to chemotherapy in several carcinomas.^31^

Two previous studies reveal that TRPC1 is highly expressed in NSCLC tissue.^32^, ^33^ In the present study, it was illustrated that TRPC1 was not only overexpressed in NSCLC tissue but also distinguished NSCLC tissue from normal tissue. Possible explanation could be that: the overexpression of TRPC1 might reflect the faster speed of cell proliferation; meanwhile, the malignant proliferation speed of NSCLC cells is faster than that of normal cells. Therefore, TRPC1 is overexpressed in NSCLC tissue.

It is illustrated that TRPC1 overexpression is linked with larger tumor size, the occurrence of lymph node metastasis and elevated TNM stage in breast cancer patients.^14^, ^15^ However, its clinical relevance with characteristics of NSCLC patients is seldom reported in the previous studies. In the current study, overexpressed TRPC1 was associated with the occurrence of LYN metastasis and high TNM stage. Possible explanations could be that: according to the evidences above, TRPC1 induces the proliferation, migration, and metastasis of tumor cell.^34^, ^35^, ^36^, ^37^ Therefore, TRPC1 is probably associated with the occurrence of metastasis and further linked with higher TNM stages in NSCLC patients.

Previous studies also investigate the prognostic value of TRPC1 in a series of solid carcinomas: TRPC1 is correlated with poorer prognosis in patients with breast cancer, colorectal cancer, and gastric cancer.^14^, ^15^, ^17^, ^18^ However, few studies report the correlation of TRPC1 with survival profile in NSCLC. In the present study, it was illuminated that elevated TRPC1 expression was associated with worse DFS in NSCLC patients, whereas TRPC1 overexpression was not associated with unsatisfying OS. Possible explanation could be that (1) TRPC1 not only induces the proliferation, migration, and metastasis of tumor cell, but also enhances the chemoresistance in several types of tumors, which might contribute to a higher relapse risk for surgical NSCLC patients.^34^, ^35^, ^36^, ^37^ Hence, TRPC1 overexpression is associated with deteriorate DFS in NSCLC patients. (2) TRPC1 overexpression is linked with the higher occurrence of metastasis. Meanwhile, TRPC1 overexpression also correlates with an advanced TNM stage as mentioned above. In addition, these unfavorable clinicopathological features could further indicate a deteriorate survival profile. Correspondingly, TRPC1 overexpression is linked with unsatisfying DFS. (3) OS would also be affected by various factors such as patients’ individual variation and the efficacy of subsequent treatment after recurrence or disease progression, etc. Consequently, TRPC1 overexpression was weakly associated with unsatisfying OS.

TRPC1 overexpression is linked with unfavorable DFS in patients receiving adjuvant chemotherapy, whereas TRPC1 expression is not correlated with DFS in those who do not receive adjuvant chemotherapy; a possible explanation could be that: patients receiving adjuvant chemotherapy are usually diagnosed with a more advanced TNM stage compared with those who do not receive adjuvant chemotherapy; thereby, the intercorrelation of TRPC1 with DFS might be amplified in NSCLC patients with the elevated TNM stage but minimized in NSCLC patients with declined TNM stage. Thus, TRPC1 overexpression is correlated with unsatisfying DFS in patients receiving adjuvant chemotherapy only. 2) TRPC1 expression is positively associated with TNM stage in NSCLC patients currently; besides, patients receiving adjuvant chemotherapy are usually diagnosed with a more advanced TNM stage compared with those who do not receive adjuvant chemotherapy; meanwhile, patients responding to adjuvant chemotherapy might reach a declined TNM stage. Thereby, TRPC1 overexpression might be correlated with unfavorable DFS by being related to TNM stage in patients receiving adjuvant chemotherapy.

Additionally, we assume that TRPC1 could serve as a potential index for prognostication in NSCLC patients. Typically, TRPC1 overexpression was linked with advanced TNM stage and LYN metastasis, which implies that TRPC1 high patients might need more progressive treatment, whereas this assumption still needs further validation in the forthcoming study.

However, some limitations existed in our study: (1) the current study was single‐center research; hence, a further control group would be desirable. (2) As a retrospective study, the section bias might exist; meanwhile, clinical data might be limited. (3) Following‐up period was comparatively short; thus, longer following‐up information was needed. (4) The upstream regulators of TRPC1 in NSCLC needed further investigation.

Collectively, TRPC1 is unregulated in NSCLC tissue with its overexpression relating to the occurrence of LYN metastasis and worse DFS in NSCLC patients, which suggests that it could serve as a potential index for prognostication in NSCLC patients.

## CONFLICT OF INTEREST

The authors of this work have nothing to disclose.

## Supporting information

Fig S1

Table S1

## Data Availability

The datasets used and/or analyzed during the current study are available from the corresponding author on reasonable request.
