# Supplementary figures and images for: Dysregulated transient receptor potential channel 1 expression and its correlation with clinical features and survival profile in surgical non‐small‐cell lung cancer patients
Source: J Clin Lab Anal. 2022 Feb 2;36(3):e24229. doi: 10.1002/jcla.24229 (PMC8906054; doi:10.1002/jcla.24229)

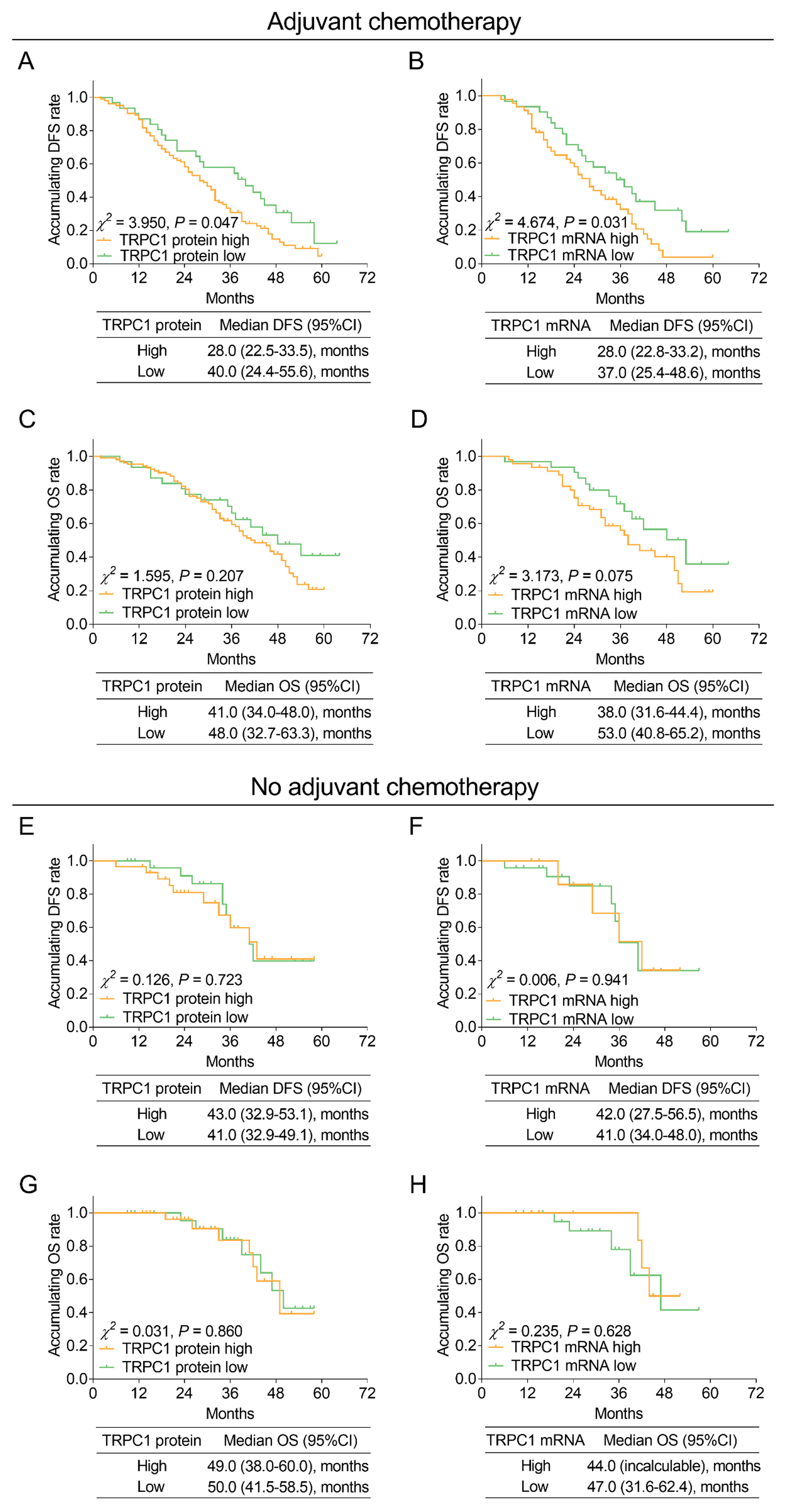

Supplement: Supplementary file 1 — Fig S1 [file JCLA-36-e24229-s002.tif]
