# Supplementary material for: Dysregulated transient receptor potential channel 1 expression and its correlation with clinical features and survival profile in surgical non‐small‐cell lung cancer patients
Source: J Clin Lab Anal. 2022 Feb 2;36(3):e24229. doi: 10.1002/jcla.24229 (PMC8906054; doi:10.1002/jcla.24229)
Supplement: Supplementary file 2 — Table S1 [file JCLA-36-e24229-s001.docx]

**Supplementary table 1.** Correlation of TRPC1 expression with tumor markers

| Items | TRPC1 IHC score | | TRPC1 mRNA expression | |
| --- | --- | --- | --- | --- |
|  | correlation coefficient (r_s_) | *P* value | correlation coefficient (r_s_) | *P* value |
| CEA | 0.077 | 0.287 | -0.028 | 0.772 |
| CA125 | -0.036 | 0.617 | -0.027 | 0.781 |

TRPC1, transient receptor potential channel 1; CEA, carcinoembryonic antigen; CA125, cancer antigen 125.
